# Dorsal attention network centrality increases during recovery from acute stress exposure

**DOI:** 10.1016/j.nicl.2021.102721

**Published:** 2021-06-09

**Authors:** T.A.A. Broeders, M.M. Schoonheim, M. Vink, L. Douw, J.J.G. Geurts, J.M.C. van Leeuwen, C.H. Vinkers

**Affiliations:** a Department of Anatomy and Neurosciences, Amsterdam Neuroscience, Amsterdam UMC, Vrije Universiteit Amsterdam, Amsterdam, The Netherlands; b Department of Psychiatry, Amsterdam Neuroscience, Amsterdam UMC, Vrije Universiteit Amsterdam, Amsterdam, The Netherlands; c Department of Psychiatry, University Medical Center Utrecht, Utrecht, The Netherlands; d Department of Experimental, Utrecht University, Utrecht, The Netherlands; e Department Developmental Psychology, Utrecht University, Utrecht, The Netherlands; f Department of Cognitive Neuroscience, Donders Institute for Brain, Cognition, and Behaviour, Radboud University Medical Center, Nijmegen, The Netherlands

**Keywords:** Acute stress, Recovery, Centrality, Functional connectivity, Dorsal attention network, Bipolar disorder

## Abstract

Stress is a major risk factor for the development of almost all psychiatric disorders. In addition to the acute stress response, an efficient recovery in the aftermath of stress is important for optimal resilience. Increased stress vulnerability across psychiatric disorders may therefore be related to altered trajectories during the recovery phase following stress. Such recovery trajectories can be quantified by changes in functional brain networks. This study therefore evaluated longitudinal functional network changes related to stress in healthy individuals (N = 80), individuals at risk for psychiatric disorders (healthy siblings of schizophrenia patients) (N = 39), and euthymic bipolar I disorder (BD) patients (N = 36). Network changes were evaluated before and at 20 and 90 min after onset of an experimental acute stress task (Trier Social Stress Test) or a control condition. Whole-brain functional networks were analyzed using eigenvector centrality as a proxy for network importance, centrality change over time was related to the acute stress response and recovery for each group. In healthy individuals, centrality of the dorsal attention network (DAN; *p* = 0.007) changed over time in relation to stress. More specifically, DAN centrality increased during the recovery phase after acute stress exposure (*p* = 0.020), while no DAN centrality change was observed during the initial stress response (*p* = 0.626). Such increasing DAN centrality during stress recovery was also found in healthy siblings (*p* = 0.016), but not in BD patients (*p* = 0.554). This study highlights that temporally complex and precise changes in network configuration are vital to understand the response to and recovery from stress.

## Highlights

• Dorsal attention network (DAN) centrality increased during stress recovery in healthy controls.
• Healthy siblings of schizophrenia patients show a similar increase during stress recovery.
• Bipolar disorder patients show no network changes during stress recovery.

## 1 Introduction

Acute stress triggers the rapid release of (nor)epinephrine, followed by a more slow release of cortisol (de Kloet et al., 2005, Joels, 2018). This integrated and dynamic response ensures the reallocation of neural resources to respond effectively to threats and restore balance afterwards (Joëls and Baram, 2009). Even though most stress responses are adaptive (van Oort et al., 2017), stress is a major risk factor for almost all psychiatric disorders (de Kloet et al., 2005). We currently do not understand what the underlying mechanisms are. A growing body of evidence suggests that not only the immediate effects of stress are important, but that an inefficient recovery from stress is a key risk factor for psychopathology (de Kloet et al., 2005, Osório et al., 2017), possibly driven by functional brain changes. In support, stress is known to dynamically affect large-scale brain networks over time (van Oort et al., 2017, Hermans et al., 2014). During stress, resources are redirected towards regions involved in detecting salient stimuli (ventral attention network; VAN) at the cost of executive functioning (frontoparietal network; FPN) (Hermans et al., 2014); potentially accompanied by increased default-mode network (DMN) activity as well (van Oort et al., 2017), a network involved in self-referential processing. During stress recovery, there is distinct resource reallocation from the acute phase, with the increase in VAN and decrease in FPN resources to be roughly reversed (Hermans et al., 2014). Furthermore, stress affects cognitive functioning across a range of cognitive domains and this might be mediated by such stress-related network reorganizations (Hermans et al., 2014, Shields, 2020, Young et al., 2017). Even though longitudinal studies exist with data before and after stress exposure (van Leeuwen et al., 2021, Reinelt et al., 2019, Quaedflieg et al., 2015), longitudinal brain network changes over time following a standardized stressor in healthy and stress-susceptible individuals has hitherto not been carried out. Investigating brain network changes after stress exposure can help identify stress-susceptible individuals and aid in our understanding why individuals are more vulnerable to stress and will go on to develop stress-related psychopathology.

In this study, we aimed to better understand how individuals recover from stress by examining changes in functional connectivity of brain networks over time before and after exposure to a standardized acute stress task (Trier Social Stress Test; TSST), and investigate how individuals with (increased risk for) psychiatric disorders do this. Recent advances in network neuroscience have highlighted the importance of more integrative network approaches (Bassett and Sporns, 2017), including network centrality which quantifies the importance of nodes within networks (Medaglia, 2017). To this end, we investigated brain network changes in the aftermath of acute stress in healthy individuals but also in two groups of more stress-susceptible individuals: healthy siblings of schizophrenia patients and euthymic bipolar I disorder (BD) patients. First-degree relatives of patients with schizophrenia are genetically at risk for a multitude of psychiatric disorders, including major depressive disorder, anxiety disorders, BD, and, inherently, schizophrenia (Cheng et al., 2018), and show heightened stress reactivity to daily stressors (Collip et al., 2011). Moreover, stress plays a major role in the onset and course of BD and schizophrenia patients (Lex et al., 2017). Rather than investigating predefined brain-regions or networks (van Leeuwen et al., 2021, Reinelt et al., 2019, Quaedflieg et al., 2015), we employed a whole-brain bottom-up approach to optimally capture relevant network centrality changes. We expected that the VAN and DMN would play a relatively more central role in the network during the initial stress response, whereas the FPN would play a less central role. We expected these changes to be roughly reversed during the subsequent stress recovery. Furthermore, we expected that these dynamic network changes over time would be more pronounced in the acute phase of stress or reduced during stress-recovery in more stress susceptible individuals: siblings of schizophrenia patients and BD patients.

## 2 Materials and methods

### 2.1 Participants

Data was acquired within two independent studies previously performed at the University Medical Center Utrecht. Study 1 (van Leeuwen et al., 2021, van Leeuwen et al., 2018a, van Leeuwen et al., 2019b) involved 40 healthy control (HC) subjects and 39 healthy siblings of patients with schizophrenia. Study 2 (van Leeuwen et al., 2019) concerned 40 HCs and 39 euthymic patients with BD. The selection of patient and sibling groups were based on data availability. Only males were included to remove known sex effects of stress on brain activity (Kirschbaum et al., 1999). Participants had neither a psychiatric disorder themselves (excluding BD for the patients) as determined by a neuropsychiatric interview (Sheehan, 1998), nor a first-degree relative with one (excluding the healthy siblings). Participants did not use corticosteroids or anti-psychotic medication, as these affect the cortisol stress response (Joels, 2018, Houtepen et al., 2015). Euthymic state of BD patients was ensured using a clinical interview (Rush et al., 1996, Young et al., 1978), defined as the absence of current depressive or manic episode. For the primary analyses of the current study, HCs of both previous studies were pooled together to increase statistical power. Formal approval was obtained for both studies from the institutional ethical review board, in accordance with the declaration of Helsinki and the guidelines for Good Clinical Practice. Written informed consent was attained from all participants.

### 2.2 Data acquisition

#### 2.2.1 General setup and stress methodology

Participants were told that they would participate in a study investigating ‘cognitive load’, all study information was given during debriefing. The TSST was used as stress intervention in the afternoon (3:15–7:00 PM) to minimize variance in diurnal cortisol secretion, and was performed as previously published (Kirschbaum et al., 1993). This specific intervention was chosen for its robust induction of a stress response (Narvaez Linares et al., 2020). Participants were randomly assigned to the stress condition or the control (no-stress) condition. The stress condition consisted of a five-minute job interview and a three-minute mental arithmetic task while facing a committee, the no-stress condition featured five minutes of free speech and a simple three-minute arithmetic task with the researcher in the same room but without a committee (Het et al., 2009). Three resting state functional scans were obtained, i.e. before (RS1), and 20 (RS2) and 90 min (RS3) after TSST onset. In between RS2 and RS3, an emotion processing and a reward task were performed inside the scanner, which was consistent across participants (van Leeuwen et al., 2018a, van Leeuwen et al., 2019b, van Leeuwen et al., 2019c). The cortisol response to the TSST was evaluated using the area under the curve related to cortisol-increase (cortisol-AUCi), meaning the area under the curve minus the area between zero and the first measurement, as previously described (van Leeuwen et al., 2018a, Pruessner et al., 2003). Additionally, the cortisol-AUCi was calculated based on the first four samples (from 10 before to 30 min after TSST onset) to reflect the acute cortisol increase. The area over the curve related to cortisol decrease was quantified using the last four samples (from 30 to 120 min after TSST onset) to reflect recovery-related cortisol depletion. Finally, a visual analog scale (VAS) was completed 10 min before, and 5 (i.e. during the TSST) and 20 min after TSST onset to assess subjective stress, and the area under the curve related to subjective stress increase (subjective-AUCi) was calculated similarly to cortisol. The difference score between consecutive measurements on the VAS characterized the acute subjective stress response and subjective stress recovery, respectively.

#### 2.2.2 Magnetic resonance imaging

All imaging was performed on Philips 3 Tesla MRI scanners (Philips Medical Systems). Whole-brain 3-dimensional T1-weighted (3D-T1) structural images were acquired using an identical sequence for both studies; repetition time (TR)/echo time (TE) = 10/4.6 ms, voxel size = 0.75 × 0.75 × 0.8 mm, flip angle = 8°. Resting-state functional magnetic resonance imaging (fMRI) was performed using 2-dimensional echo planar imaging-sensitivity encoding (EPI-SENSE) sequences: study 1 (TR/TE = 2000/35 ms, voxel size = 2.7 × 2.7 × 3.4 mm, flip angle = 72.5°, gap = 0.43 mm, *N*volumes = 202, scan time = 7 min) and study 2 (TR/TE = 1600/23 ms, voxel size = 4 mm isotropic, flip angle = 70°, gap = 0.40 mm, *N*volumes = 300, scan time = 8:13 min).

### 2.3 Data processing

#### 2.3.1 MRI preprocessing

Data preprocessing was performed using tools from the FMRIB (Functional Magnetic Resonance Imaging of the Brain) software library (FSL 5.0.9, http://www.fmrib.ox.ac.uk/fsl). For 3D-T1 images brain extraction, white matter and grey matter segmentation was performed, followed by segmentation of deep grey matter (DGM) regions and a nonlinear registration to 2 mm standard-space (MNI-152). All three fMRI scans were preprocessed by performing motion-correction, brain extraction, smoothing (5 mm isotropic kernel), linear boundary-based co-registration, and subsequent automatic identification and removal of motion artifacts using ICA-AROMA. Thereafter, regression of white matter and cerebrospinal fluid signal, nonlinear high-pass temporal filtering, linear boundary-based registration to 3D-T1 images and finally co-registration and resampling to standard space at 4 mm was performed. Participants that moved too much (i.e. root mean squared relative displacement of > 0.3 mm) during at least one of the post-stress MRI scans were excluded from further analysis, resulting in the exclusion of three BD patients in the no-stress condition. For a detailed description of preprocessing steps, see the supplementary material. A distortion mask was created for the entire subject sample to ensure comparability between subjects, excluding non-reliable fMRI signal (based on the robust range of signal intensity) and non-grey matter voxels.

#### 2.3.2 Network centrality

**Fig. 1:**
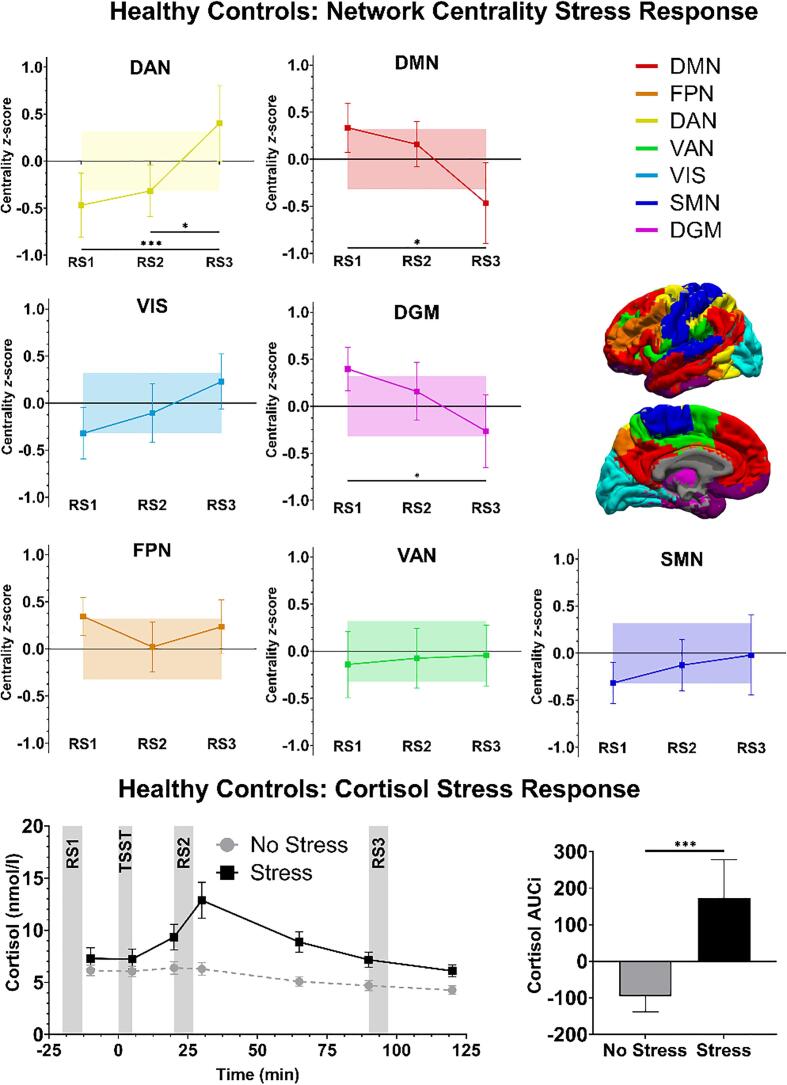
Healthy participants in the stress condition showed a significant change in eigenvector centrality of the dorsal attention network (DAN), default-mode network (DMN) and deep grey matter (DGM) over time. Further investigating these three networks revealed a significant increase in DAN centrality during the recovery from acute stress exposure. The lines represent the centrality for each network of the stress condition relative to the no-stress condition, and error bars represent the 95% confidence intervals (CIs). The colored surfaces correspond to the 95% CIs for the no-stress condition, which by definition have a mean of zero and a standard deviation of one. The bottom graphs shows the cortisol response to the Trier Social Stress Test (TSST), as the area under the curve of cortisol increase (Cortisol AUCi) was higher in the stress condition than the no-stress condition. RS1 = pre-stress exposure, RS2 = 20 min. post-exposure, RS3 = 90 min. post-exposure, FPN = frontoparietal network, VAN = ventral attention network, VIS = visual network, SMN = sensorimotor network, *p-value < 0.05, ***p-value < 0.001.

Voxel-wise eigenvector centrality mapping (ECM) was performed for voxels within the distortion mask for each participant using fastECM (Wink et al., 2012). Eigenvector centrality not only considers its direct connections, which is done to calculate degree for instance, but also attributes additional weight to connections with nodes that are connected more strongly (i.e. more central) themselves (Wink et al., 2012) and allows for a voxel-wise approach that captures the large-scale effects of stress, as previously proposed (Reinelt et al., 2019). We utilized the spatially hierarchical organization of the brain (Yeo et al., 2011) by investigating changes on the level of resting-state networks first and, post-hoc, zooming in to find regional changes. Resting-state networks were defined using a literature-based seven-network cortical parcellations atlas (Yeo et al., 2011) and a DGM network was based on DGM segmentations performed on controls using FIRST. Networks consisting of at-least 30% of voxels with sufficient-quality grey matter signal were used, which resulted in the exclusion of the limbic network. A regional atlas was used to allow within-network parcellations of individual brain regions, based on the Brainnetome atlas (Fan et al., 2016) with its DGM regions replaced by FIRST segmentations. The network atlas was superimposed on this regional atlas, all brain regions were assigned to the network with which they showed most overlap. For regional analyses, only regions that overlapped most strongly with one of the included networks and consisted of at-least 30% non-distorted voxels were analyzed. In the end, we analyzed the DMN (36 brain regions), FPN (21 brain regions), dorsal attention network (DAN, 25 brain regions), VAN (23 brain regions), visual network (21 brain regions), sensorimotor network (33 brain regions), and DGM network (14 brain regions; see Fig. 1). Centrality was averaged over all voxels within the network and brain-region masks, used for statistical analysis of network and regional centrality, respectively. Finally, centrality values were transformed to z-scores based on the distribution of healthy controls in the no-stress condition. We have used slightly different network definitions from previous studies to be able to parcellate the whole brain into distinct networks (Hermans et al., 2014, van Leeuwen et al., 2021); most importantly, the current FPN overlaps most strongly with the previous definition of the executive control network and the VAN with the definition of the salience network.

#### 2.3.3 Network connectivity

To investigate whether a change in centrality reflected altered connectivity between specific networks or within the network itself the same preprocessed data was used as for the centrality computation. Functional connectivity matrices were computed, based on Pearson correlations between all pairs of brain regions. Fisher’s Z-transformation was applied to these matrices to attain normally distributed connectivity values. Thereafter, all correlations were made absolute and divided by mean connectivity for each subject separately, creating a whole-brain matrix of relative connectivity. Subsequently, columns of the matrix from regions belonging to the same network were averaged, i.e. within-network connectivity. Similarly, connectivity between a network and all other networks were calculated by averaging values of all respective connections, resulting in six between-network connectivity values. Thus, in total, seven connectivity values could be obtained per network per participant.

### 2.4 Statistical analyses

The statistical analyses were performed in SPSS (release 26.0.0.1). Normality was checked using histogram inspection and the Kolmogorov-Smirnov test. Post-hoc pairwise comparisons were performed using estimated marginal means. False discovery rate (fdr) adjusted *p*-values were calculated with *p.adjust* in R (version 3.6.1) to correct for testing across multiple networks, and values below 0.05 were considered significant.

One-way ANOVAs and chi-squared tests were used to compare participant characteristics and the average movement in the MRI scanner between groups for each study, as well as to compare HC groups of both studies. Subsequently, cortisol- and subjective-AUCi levels compared using linear mixed models (LMMs) with a 2 × 3 design per study to compare groups (condition × group) and with a 2 × 2 design to compare HCs per study (condition × study).

To identify network changes related to stress in healthy controls, discovery analyses were run per network using LMMs on network centrality z-scores by pooling HCs from both studies to increase statistical power. An interaction effect was indicative of a relation to the stress response. The study of acquisition had been added as covariate. Firstly, analyses were run using a 2 × 3 design (condition × time) for each network, with a significant interaction effect (using uncorrected p-values) being used to identify networks that were related to the stress exposure. Subsequently, LMMs were used on these networks at RS1 (2 × 1, condition × time) to compare groups before stress exposure, and at RS1-RS2 and RS2-RS3 (2 × 2, condition × time) to evaluate change related to the initial stress response and to stress recovery, respectively. Subsequently, the HCs were investigated per study, to see whether effects related to the initial stress response or stress recovery were consistent across both studies.

To better understand connectivity changes observed during the initial stress response or stress recovery, firstly, Pearson’s correlation was calculated between centrality change scores (i.e. RS2-RS1 and RS3-RS1) and the cortisol/subjective stress response. Next, regional centrality and within- and between-network connectivity were investigated per network using 2 × 2 LMMs (condition × time).

To identify the clinical relevance of our findings, the centrality of the networks that related to stress in HCs were investigated in healthy sibling and BD patient groups as well. LMMs were ran per group over all timepoints to see whether centrality changed dissimilarly over time between conditions (2 × 3, condition × time). Thereafter, centrality at RS1 was used to identify differences between conditions before stress exposure (2 × 1, condition), and at RS1-RS2 and RS2-RS3 to investigate the initial stress response and stress recovery, respectively (2 × 2, condition × time). Finally, models were performed using three-way interactions (condition × time × group) to identify potential differences between groups.

## 3 Results

### 3.1 Participant characteristics

No significant differences were found between the BD patient (*N* = 36) and healthy sibling (*N* = 39) groups compared to HCs (*N* = 80) related to participant characteristics (see Table 1; see Supplementary Table 1 for details per condition). HCs from the two studies did differ with regards to their age (*F*(1,78) = 9.76, *p* = 0.003) and BMI (*F*(1,78) = 4.50, *p* = 0.037).

**Table 1:** Participant characteristics.

	Study 1			Study 2			Controls
	Healthy Controls (n = 40)	Healthy Siblings (n = 39)	Group Difference	Healthy Controls (n = 40)	BD Patients (n = 36)	Group Difference	Group Difference
Age, years	33.9 (±8.7)	33.2 (±9.1)	*F* = 0.13 *p* = 0.723	39.7 (±7.5)	40.9 (±8.5)	*F* = 0.47 *p* = 0.494	***F* = 9.76** ***p* = 0.003**
Education^¥^, level obtained	7 (5–8)	7 (5.75–8)	*F* = 0.16 *p* = 0.693	6 (4–6)	6 (5–7)	*F* = 2.73 *p* = 0.103	–
Handedness, right/left/both	37/3/0	31/6/2	*X^2^* = 3.52 *p* = 0.172	37/3/0	31/5/0	*X^2^* = 0.82 *p* = 0.365	*X^2^* = 0.00 *p* = 1.000
Smoking, yes/no	8/32	12/27	*X^2^* = 1.21 *p* = 0.271	6/34	8/28	*X^2^* = 0.66 *p* = 0.417	*X^2^* = 0.35 *p* = 0.556
Body Mass Index, kg/m^2^	24.3 (±2.4)	24.4 (±3.4)	*F* = 0.05 *p* = 0.817	25.8 (±3.6)	25.7 (±3.3)	*F* < 0.01 *p* = 0.995	***F* = 4.50** ***p* = 0.037**
Underwent MRI before, yes/no	22/18	13/26	*X^2^* = 3.76 *p* = 0.053	24/16	21/15	*X^2^* = 0.02 *p* = 0.883	*X^2^* = 0.21 *p* = 0.651
Movement in MRI	0.6 (±0.03)	0.06 (±0.2)	*F* = 0.02 *p* = 0.880	0.08 (±0.02)	0.10 (±0.04)	***F* = 7.15** ***p* = 0.009**	***F* = 8.12** ***p* = 0.006**

*Note.* The descriptive characteristics were compared between the four groups of both studies. All values represent means and standard deviations for continuous variables, the values represent medians and interquartile range (^¥^) or frequencies for categorical variables. Education represents the highest level of education attained in the Dutch system (Study 1: 1–8, Study 2: 1–7), and was not compared between control groups of both studies due to the dissimilar scaling. Movement in MRI scanner is based on the mean framewise displacement between consecutive volumes. BD = Bipolar disorder.

### 3.2 Stress response

**Fig. 2:**
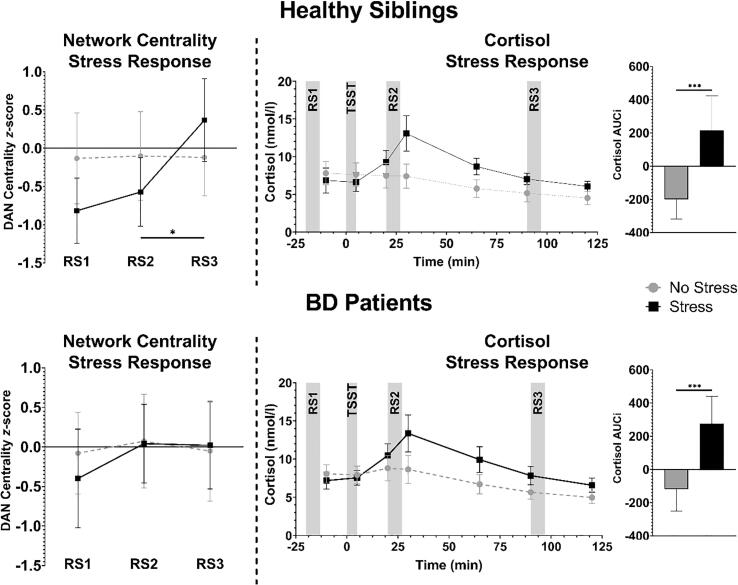
Healthy siblings of schizophrenia patients showed a significant change in eigenvector centrality of the dorsal attention network (DAN) over time after acute stress exposure, but no change was observed in bipolar disorder (BD) patients. The cortisol stress response shows the efficacy of the Trier Social Stress Test (TSST) to induce stress in the stress condition of both groups, as indicated by the area under the curve of cortisol increase (Cortisol AUCi) which was higher in the stress condition than the no-stress condition for both groups. RS1 = pre-stress exposure, RS2 = 20 min. post-exposure, RS3 = 90 min. post-exposure, *p-value < 0.05, ***p-value < 0.001.

The stress condition resulted in a robust cortisol stress response in all groups (condition: *F*(1,148) = 51.32, *p* < 0.001), as characterized by higher cortisol-AUCi in the stress condition. This response was not different between groups (group: *F*(2,148) = 0.54, *p* = 0.584; group × condition: *F*(2,148) = 1.08, *p* = 0.343). Additionally, although cortisol-AUCi was higher in HCs from study 2 compared to study 1 (study: F(1,75) = 6.40, *p* = 0.014), the cortisol stress response was not different between studies (study × condition: F(1,75) = 1.28, *p* = 0.261). Thus, we did not find any differences between the cortisol stress response in HCs (see Fig. 1) compared to healthy siblings and BD patients (see Fig. 2), as previously published (van Leeuwen et al., 2018a, van Leeuwen et al., 2019c).

Similarly, all groups showed a robust subjective stress response (condition: F(1,148) = 19.95, *p* < 0.001) that was not different between groups (group: F(2,148) = 1.232, *p* = 0.295; group × condition: F(1,148) = 0.67, *p* = 0.516) and the subjective-AUCi stress response was consistent across healthy controls from both studies (study: F(1,73) = 0.05, *p* = 0.882; study × condition: F(1,73) = 0.13, *p* = 0.718).

### 3.3 MRI scanner movement

A difference was found in the amount of movement in the MRI scanner between HCs of both studies (*F*(1,78) = 8.12, *p* = 0.006), with HCs from study 2 moving more compared to HCs from study 1. BD patients moved more than HCs (*F*(1,74) = 7.15, *p* = 0.009), but healthy siblings did not differ from HCs (*F*(1,77) = 0.02, *p* = 0.880).

### 3.4 Brain network stress response in healthy individuals

Across the three resting state scans, centrality changed over time for HCs in the acute stress exposure relative to the no-stress condition in the DMN (condition × time: *F*(2,156) = 3.66, *p* = 0.028), DAN (condition × time: *F*(2,156) = 5.07, *p* = 0.007), and DGM (condition × time: *F*(2,156) = 3.96, *p* = 0.021), indicating altered connectivity changes following stress in those networks over time. In contrast, this was not the case for the other four networks (all *p*-values ≥ 0.069; see Table 2). We therefore further scrutinized the DMN, DAN, and DGM in more detail. In the DMN, DAN and DGM, network centrality was not significantly different at RS1 between HCs in the stress condition compared to the no-stress condition (condition: DMN, *F*(1,78) = 2.63, p_fdr_ = 0.109; DAN, *F*(1,78) = 4.21, p_fdr_ = 0.070; DGM, *F*(1,78) = 4.09, p_fdr_ = 0.070). Between RS1 and RS2 (i.e. before and 20 min after the experimental condition), exposure to the stress and control condition did not result in significantly different trajectories of network centrality in any of the networks (DMN, DAN, and DGM: condition × time: all *p_fdr_-*values *=* 0.626). Between RS2 and RS3 (i.e. the recovery phase), DAN centrality change over time was different between the stress and the control condition (condition × time: *F*(1,78) = 7.74, *p_fdr_* = 0.020), but not for the DMN (condition × time: *F*(1,78) = 4.60, *p_fdr_* = 0.053) or DGM (condition × time: *F*(1,78) = 2.67, *p_fdr_* = 0.106) (Fig. 1). This indicates that for the DAN, centrality changed during the recovery phase following acute stress exposure.

**Table 2:** Network centrality of controls in the stress condition relative to the no-stress condition.

**Healthy Controls**	Stress-group Relative to no-stress group			Mixed Effects Model Stress vs. no-stress					
	Centrality z-score Mean (±SD)			Time × Condition		Condition		Time × Condition	
	RS1	RS2	RS3	RS1-RS2-RS3		RS1		RS1-RS2	RS2-RS3
**Brain Network**									
DMN	0.334 (±0.82)	0.158 (±0.75)	−0.467 (±1.35)	*F* = 2.63 *p* = 0.028		*F* = 2.63 *p_fdr_* = 0.109		*F* = 0.38 *p_fdr_* = 0.626	*F* = 4.60 *p_fdr_* = 0.053
FPN	0.345 (±0.63)	0.021 (±0.82)	0.236 (±0.88)	*F* = 3.49 *p* = 0.418		*–*		*–*	*–*
DAN	−0.468 (±1.07)	**−0.317 (±0.85)**	**0.402 (±1.26)**	*F* = 3.24 *p* = 0.007		*F* = 4.21 *p_fdr_* = 0.070		*F* = 0.24 *p_fdr_* = 0.626	***F* = 7.74** ***p_fdr_* = 0.020**
VAN	−0.140 (±1.10)	−0.073 (±0.99)	−0.044 (±1.01)	*F* = 0.36 *p* = 0.937		*–*		*–*	*–*
VIS	−0.321 (±0.86)	−0.106 (±0.98)	0.228 (±0.92)	*F* = 2.36 *p* = 0.069		*–*		*–*	*–*
SMN	−0.317 (±0.68)	−0.127 (±0.85)	−0.022 (±1.21)	*F* = 2.74 *p* = 0.508		*–*		*–*	*–*
DGM	0.396 (±0.72)	0.158 (±0.96)	−0.265 (±1.21)	*F* = 4.09 *p* = 0.021		*F* = 4.09 *p_fdr_* = 0.070		*F* = 0.97 *p_fdr_* = 0.626	*F* = 2.67 *p_fdr_* = 0.106

*Note.* Healthy controls (HCs) in the stress condition show an increase over time in dorsal attention network (DAN), centrality between RS2 and RS3, relative to distribution of HCs in the no-stress condition. The *z*-scores represent values of HCs in the stress condition relative to the distribution of HCs in the no-stress condition. DMN = default-mode network, FPN = frontoparietal network, VAN = ventral attention network, VIS = visual network, SMN = sensorimotor network, DGM = deep grey matter, RS1 = pre-stress exposure, RS2 = 20 min. post-exposure, RS3 = 90 min. post-exposure, p_fdr_ = *p*-values are corrected for multiple comparisons using the false discovery rate.

Based on these findings, we examined DAN centrality during the recovery phase in more detail. A positive correlation was found between DAN centrality change during the recovery phase to recovery-related cortisol depletion (*r*(77) = 0.25, *p* = 0.024) and not acute cortisol increase (*r*(77) = 0.14, *p* = 0.208), further suggesting a link between DAN centrality change and stress recovery. However, no relation between DAN centrality change scores and the acute subjective stress response (*r*(75) = 0.14, *p* = 0.298) or subjective stress recovery (*r*(75) = -0.12, *p* = 0.298) was found. Movement in the MRI also did not correlate to DAN centrality change scores (r(79) = 0.11, *p* = 0.344), thus although movement differed between studies it is not expected to have a confounding effect on the results. Zooming in on the individual brain regions most strongly overlapping with the DAN, centrality changes were most notable in area 5 (condition × time: left lateral, *F*(1,78) = 8.09, *p_fdr_* = 0.028; right medial, *F*(1,78) = 12.0, *p_fdr_* = 0.014) and area 7 (condition × time: left rostral, *F*(1,78) = 9.09, *p_fdr_* = 0.028); left intraparietal, *F*(1,78) = 11.5, *p_fdr_* = 0.014; right medial, *F*(1,78) = 8.31, *p_fdr_* = 0.028) (see Supplementary Table 2). No changes in connectivity over time were found both for within- (i.e. DAN-DAN) and between network DAN connectivity (i.e. DAN-DMN, DAN-FPN, etc.) (all *p_fdr_-*values*=>*0.220) (see Supplementary Table 3).

Stratified analyses for DAN centrality during stress recovery in HCs from study 1 and 2 showed comparable results to the grouped analysis; as there was no difference between conditions at RS1 (i.e. before stress exposure) (condition: study 1, *F*(1,38) = 3.76, *p* = 0.060; study 2, *F*(1,38) = 0.72, *p* = 0.401); there was no difference in centrality change over time between RS1 and RS2 (condition × time: study 1, *F*(1,38) = 0.54, *p* = 0.465; study 2, *F*(1,38) = 0.02, *p* = 0.904), signifying no DAN centrality changes during the initial stress response; and centrality change between RS2 and RS3 (i.e. the recovery phase) showed comparable directionality to the grouped analyses (condition × time: study 1, *F*(1,38) = 3.62, *p* = 0.065; study 2, *F*(1,38) = 4.27, *p* = 0.046).

### 3.5 Healthy siblings

We then analyzed DAN centrality in at risk healthy siblings of schizophrenia patients. Across all three resting state scans, change of DAN centrality over time was different between the stress and no-stress conditions (condition × time: *F*(2,74) = 4.32, *p* = 0.017). DAN centrality did not change dissimilarly between the stress and no-stress conditions between RS1 and RS2 for at risk individuals (condition × time: *F*(1,37) = 0.20, *p* = 0.657). Between RS2 and RS3, however, there was a significant interaction effect of condition and time (condition × time: *F*(1,37) = 6.35, *p* = 0.016), showing a significantly increased DAN centrality following stress recovery (*p_fdr_* = 0.003) but not in the control condition (*p_fdr_* = 0.944) (see Fig. 2, top). At RS1, DAN centrality was not significantly different between the stress and control condition (condition: *F*(1,37) = 3.77, *p* = 0.060; see Table 3). Together, these findings indicate similar DAN centrality increases during recovery following acute stress exposure as in healthy individuals that are not at risk.

**Table 3:** Network eigenvector centrality change of schizophrenia siblings and bipolar patients in the stress condition compared to siblings and patients in the no-stress condition.

	DAN Centrality Relative whole no-stress group			Mixed Effects Model Stress vs. No-Stress		
	Centrality z-score Mean (±SD)			Condition	Time × Condition	
	RS1	RS2	RS3	RS1	RS1-RS2	RS2-RS3
**Study 1**						
Siblings						
No-Stress	−0.134 (±1.27)	−0.101 (±1.24)	−0.120 (±1.08)	*F* = 3.77 *p* = 0.060	*F* = 0.12 *p* = 0.727	***F* = 6.35** ***p* = 0.016**
Stress	−0.817 (±0.89)	**−0.572 (±0.93)**	**0.368 (±1.13)**			
Controls						
No-Stress	−0.090 (±1.23)	0.101 (±1.02)	−0.029 (±1.07)	*F* = 3.76 *p* = 0.060	*F* = 0.54 *p* = 0.465	*F* = 3.62 *p* = 0.065
Stress	−0.789 (±1.04)	−0.249 (±0.95)	0.422 (±1.41)			
Between-group differences				*–*	*–*	*F* = 0.04 *p* = 0.957

**Study 2**						
Patients						
No-Stress	−0.080 (±1.00)	0.072 (±1.15)	−0.052 (±1.23)	*F* = 0.52 *p* = 0.477	*F* = 0.20 *p* = 0.657	*F* = 0.36 *p* = 0.554
Stress	−0.400 (±1.21)	0.042 (±0.97)	0.020 (±1.07)			
Controls						
No-Stress	0.090 (±0.72)	−0.101 (±1.02)	0.029 (±1.07)	*F* = 0.72 *p* = 0.401	*F* = 0.02 *p* = 0.904	***F* = 4.27** ***p* = 0.046**
Stress	**−**0.148 (±1.03)	**−0.385 (±0.95)**	**0.383 (±1.12)**			
Between-group differences				*–*	–	*F* = 0.72 *p* = 0.400

*Note.* Siblings in the stress condition show an increase over time in dorsal attention network (DAN) centrality between RS2 and RS3, relative to siblings in the no-stress condition. Bipolar disorder patients in the stress condition do not show such a change. The *z*-scores represent values of siblings/patients in the stress condition relative to the distribution of siblings/patients in the no-stress condition. All *p*-values are corrected for multiple comparisons using the false discovery rate per group. RS1 = pre-stress exposure, RS2 = 20 min. post-exposure, RS3 = 90 min. post-exposure.

No relation was found between DAN centrality change scores during the recovery phase and recovery-related cortisol depletion (*r*(37) = -0.03, *p* = 0.873) or acute cortisol increase (*r*(37) = -0.71, *p* = 0.666). This means that DAN centrality change did not relate to cortisol increase during the recovery phase, as was found in HCs.

### 3.6 BD patients

We next analyzed DAN centrality in BD patients. Inspecting DAN centrality changes across all three scans, revealed that DAN centrality change over time was not different between conditions (condition × time: *F*(2,68) = 0.43, *p* = 0.653). Accordingly, DAN centrality change between RS1 and RS2 (i.e. the initial stress response) did not differ between the stress and no-stress conditions (condition × time: *F*(1,34) = 0.12, *p* = 0.727). Between RS2 and RS3, no significant differences in DAN centrality change over time was found between stress and no-stress (condition × time: *F*(1,34) = 0.36, *p* = 0.554), suggesting no DAN centrality change related to stress recovery in these patients (see Fig. 2, bottom). Furthermore, DAN centrality was not different between conditions at RS1 (condition: *F*(1,34) = 0.52, *p* = 0.477), indicating that centrality of this network was not different between both before stress exposure.

Similarly to the siblings, no relation was found between DAN centrality change scores for the recovery phase and recovery-related cortisol depletion (*r*(34) = -0.10, *p* = 0.567) or acute cortisol increase (*r*(34) = 0.17, *p* = 0.335).

It should be noted that a group × time × condition interaction effect to compare the DAN centrality stress response during the recovery phase between all three groups was not significant (*F*(2,149) = 0.01, *p* = 0.994).

## 4 Discussion

This study investigated changes in functional brain networks related to acute stress exposure. We show that in healthy individuals DAN centrality increased during the recovery phase following acute stress exposure but not in other brain networks (including the DMN, FPN, VAN, visual-, sensorimotor-, and DGM network). This increasing DAN centrality was particularly related to the extent of cortisol depletion during the recovery phase. Healthy siblings of schizophrenia patients show a similar increase in DAN centrality during stress recovery, but BD patients do not show such changes in DAN centrality.

The DAN is a higher-order network that is particularly involved in the top-down process of externally oriented actions and perceptions (Liu et al., 2017). As such, increasing DAN centrality in the aftermath of stress might indicate that focusing attention on external stimuli becomes more important while recovering from an acute stressor. Previous work has shown that stress is related to more attentional vigilance and less deliberate processing of attentional stimuli (Hermans et al., 2014, Qi et al., 2018), while intrinsic attentional control was reduced between 20 and 40 min after stress exposure (Sanger et al., 2014). The upregulation of DAN centrality might, therefore, be related to an amplification of attentional control. Moreover, the reduction of attentional control during the acute stress phase was shown to be particularly present in conditions containing orientational distractors, and stressed participants showed more difficulty with suppressing spatial distractors in particular (Sanger et al., 2014). Interestingly, we have found that the increase of DAN centrality was most prominent in DAN regions located in the parietal cortex (i.e. in Brodmann’s regions 5 and 7), which are also more strongly involved in recognition of spatial location and orientation. In addition to attention, stress can affect working memory and reward processing as well (Shields, 2020) and regions of the DAN were also involved in these processes (Majerus et al., 2018, Taylor et al., 2004). On top of that, aberrant DAN connectivity has been related to the severity of symptoms in patients with post-traumatic stress disorder (Sheynin et al., 2020, Zhu et al., 2020). These findings highlight the possible role of the DAN in stress-induced changes of several cognitive functions, which warrants further research. Caution for inverse inferences are needed, however, as resting-state activity was acquired and links to ongoing cognitive processes are speculative. Finally, as DAN centrality increase during stress recovery related to the amount of cortisol reduction during recovery and not to subjective stress recovery, and stress recovery is heavily linked to cortisol (de Kloet et al., 2005, Joels, 2018), future studies are indicated to study the specific effects of cortisol on the DAN.

Stress recovery is thought to involve a reversal of the changes that occurred in the acute stress phase, hence affecting the same networks (Hermans et al., 2014). Our results corroborate the added value of using a bottom-up approach with the implication of a network that has not been reported in relation to stress recovery before, the DAN. This shows that network recovery from stress may constitute of a more complex reconfiguration of functional connectivity than originally postulated (Hermans et al., 2014). DAN centrality did not significantly change during the initial stress response, which contrasts the notion that initial network changes are reversed during the recovery phase. Finally, contrary to our expectations, we did not capture changes during the initial stress response in the VAN, DMN and FPN. This might be due to slightly different definition of the networks. However, regions within the DAN that showed the largest effects were those located near the anatomical borders with the SMN and visual network, making such an explanation less likely. Alternatively, a recent study has shown that FPN regions acted as connector to other networks while within-network connectivity was reduced during acute stress, and the opposite was found for the DMN (Zhang et al., 2020). This might mean that overall centrality of such networks does not change, hence the null result in this study. The connectivity between regions of the DAN and regions of the VAN, DMN and FPN were evaluated in the current study as well, however, and these did not seem to be related stress recovery as well.

With regards to BD patients, no increase in DAN centrality during stress recovery was present, hinting that the network might be less dynamic in BD patients and thereby predisposing patients to stress-induced symptomatology. Moreover, previous research has indicated that BD patients show DAN hyperconnectivity during manic- compared to euthymic states (Brady et al., 2017), therewith further supporting the importance of the DAN functioning in BD symptomatology. However, it is worth considering that the lack of DAN centrality change after stress exposure in euthymic BD patients could indicate an adaptive response, as these patients are currently in a stable state. Previous research suggests that glucocorticoid receptor functioning is reduced in BD patients (Belvederi Murri et al., 2016) possibly driving a reduced responsiveness to cortisol increase. This might explain why the BD patients showed a robust cortisol stress response, but no DAN centrality increase.

Healthy siblings of schizophrenia patients showed a normal DAN-related stress recovery. Previous work on connectivity changes post-stress with the data from study 1 showed upregulation of VAN connectivity at rest following acute stress (van Leeuwen et al., 2021) and deactivation in key DMN and VAN regions during an emotional task (van Leeuwen et al., 2018) in controls, but not in the healthy sibling group. Those studies suggest that these siblings exhibit altered connectivity after acute stress. Unaltered centrality change over time in healthy siblings does not preclude changes in other network properties, but supports that longitudinal dynamics of network centrality post-stress is preserved. Additionally, DAN-related stress recovery was not related to cortisol stress recovery in siblings, suggesting that stress regulation might be altered in these individuals.

Our study has inherent limitations. Firstly, eigenvector centrality provides a relative value (Wink et al., 2012), herewith correcting for global variations in functional connectivity known to affect comparisons between groups (Finn et al., 2015). This is important as patients with BD and schizophrenia were reported to have lower global connectivity (Argyelan et al., 20142014). Future studies should consider how stress affects the functional brain network across hierarchical scales (Medaglia, 2017) and how the network dynamically changes over time (i.e. using dynamic functional connectivity) (Preti et al., 2017). Furthermore, in light of the relevance of sex-specific effects for stress (Kirschbaum et al., 1999), our inclusion of only male participants reduces the generalizability of the current findings, which requires further investigation. Additionally, diurnal variations in cortisol levels were accounted for by investigating all participants around the same time of day, but seasonal variations were not investigated (Hansen et al., 2008). Another limiting factor is that functional networks which did not significantly relate to stress in HCs might have been changing over time in the BD patient and healthy sibling groups, this is not captured with our statistical analysis. Larger sample sizes are needed for these at-risk groups in particular, to further investigate the network stress recovery across multiple networks in these groups. Relatedly, not being able to include the limbic network due to scanner distortion and the cerebellum as it is largely not within the field of view during scanning limits our results, as these might be important for the stress response as well. Finally, even though siblings of schizophrenia patients are at risk of a range of mood disorders (Cheng et al., 2018), BD siblings would have made a more clear-cut comparison because of a more similar genetic profile.

In conclusion, this study showed that recovery from acute stress exposure in healthy individuals involves a dynamic increase of DAN centrality over time that is related to the cortisol stress response. Such longitudinal changes in DAN centrality during the aftermath of stress were observed in siblings of schizophrenia patients as well, but were absent in patients with BD, possibly indicative of reduced DAN dynamics related to stress recovery. This study adds to the existing evidence that temporally complex changes in network configuration are vital to understand the response to and recovery from stress exposure.

## CRediT authorship contribution statement

**T.A.A. Broeders:** Writing - original draft, Methodology, Formal analysis. **M.M. Schoonheim:** Methodology, Writing - review & editing, Supervision. **M. Vink:** Conceptualization, Project administration. **L. Douw:** Methodology, Writing - review & editing. **J.J.G. Geurts:** Writing - review & editing. **J.M.C. van Leeuwen:** Conceptualization, Investigation, Writing - review & editing. **C.H. Vinkers:** Conceptualization, Writing - review & editing, Supervision, Project administration, Funding acquisition.
