# Supplementary material for: Dorsal attention network centrality increases during recovery from acute stress exposure
Source: Neuroimage Clin. 2021 Jun 9;31:102721. doi: 10.1016/j.nicl.2021.102721 (PMC8214139; doi:10.1016/j.nicl.2021.102721)
Supplement: Supplementary Data 1 [file mmc1.docx]

**Supplementary Material**

**Detailed MRI preprocessing**

**Anatomical volume.** Data preprocessing was predominantly performed using in-house written scripts, integrating tools from the FMRIB (Functional Magnetic Resonance Imaging of the Brain) software library (FSL 5.0.9, http://www.fmrib.ox.ac.uk/fsl). Firstly, brain extraction was performed on the 3D T1-weighted anatomical volume after image bias reduction and residual neck voxel removal, using a fractional intensity threshold of 0.1 in BET. This was followed by segmentation into white-matter (WM), grey-matter (GM), and cerebrospinal-fluid (CSF) using FAST. Both these steps were performed using the SIENAX pipeline. Subsequently, segmentation of deep GM (DGM) was performed using FIRST. Finally, a nonlinear registration to 2mm standard-space (MNI-152) was computed using initial affine registration of the non-brain-extracted volume with FLIRT, which was required for nonlinear registration of the brain-extracted volume using FNIRT.

**Resting-state scans.** The 4D resting state images for all three runs of each participant were processed using the FEAT pipeline, including motion-correction by rigid-body registrations to the middle volume using MCFLIRT. Brain extraction, using BET, was performed to create a brain-mask for the mean volume of the fMRI data. This was followed by Gaussian spatial smoothing using a 5mm isotropic kernel using SUSAN. Thereafter, a linear boundary-based co-registration transformation matrix was calculated for transforming the middle volume to the 3D-T1w image, but scans were still kept in subject space. The next step involved creating independent components using MELODIC, followed by implementing the ICA-AROMA algorithm which automatically identifies and removes components that represent movement. Subsequently, regression of the WM and CSF signal was performed by removing the DGM segmentations, from the WM and CSF masks. On top of that, extra-cortical CSF signal was removed to ensure that no cortical GM signal was accidentally regressed-out. A ventricle-mask in standard-space was transformed to subject-space by inversely applying the previously created transformation matrix using nearest neighbor interpolation, no threshold was applied in this step to ensure inclusion of all ventricle signal. Then voxels from the CSF-mask were removed that were not included in the ventricle-mask, resulting in a CSF mask that excludes CSF around the cortex. Nonlinear high-pass temporal filtering was applied using a 50-second cut-off. Finally, the filtered and processed resting-state scans were nonlinearly registered to 4mm standard-space using formerly established transformation parameters.

**Distortion Mask.** A distortion mask that excludes non-reliable fMRI signal and non-grey matter voxels was created, to be used during eigenvector centrality mapping. To achieve this, the SIENAX-based GM mask was merged with the FIRST-based DGM region masks. This binary mask was then non-linearly transformed to 2mm standard-space, using transformation matrices created during previous registration steps and nearest-neighbor interpolation. In standard-space, the GM masks for every participant were summed and thresholded at >25% of the number of participants. This resulted in a relatively thick GM estimation, ensuring inclusion of a large portion of possible grey matter signal. Next, the resulting GM-mask was subsampled to 4mm isotropic voxel size.

In addition, a subject-space mask was created for each individual subject in order to exclude the effects of distortion by removing the lowest quartile of the robust signal-intensity range, which represents unreliable fMRI signal. These individual fMRI masks were registered to standard space and temporally concatenated, followed by voxel-wise temporal mean thresholding at >90%. This made sure that variance in scan coverage was minimized and thus that each functional connectome had a comparable size. Finally, both the GM-mask and the fMRI-mask were combined.

**Supplementary Table 2.** Participant characteristics per condition.

| **Study 1** | Healthy Controls | | Healthy Siblings | |  | Group difference | | |
| --- | --- | --- | --- | --- | --- | --- | --- | --- |
|  | No-Stress  (N=20) | Stress  (N=20) | No-Stress  (N=20) | Stress  (N=19) |  | Test-statistic | p-value |  |
| Age, years | 33.1±8.5 | 34.8±9.1 | 33.9±10.8 | 32.5±7.4 |  | *F*=0.24 | 0.872 |  |
| Education^¥^,  level obtained | 7 (7-8) | 7 (5-8) | 7 (5-8) | 7 (6-8) |  | *F*=0.55 | 0.648 |  |
| Handedness, right/left/both | 18/2/0 | 19/1/0 | 14/4/2 | 17/2/0 |  | *X^2^*=8.81 | 0.184 |  |
| Smoking, yes/no | 1/19 | 7/13 | 6/14 | 6/13 |  | *X^2^*=5.98 | 0.112 |  |
| Body Mass Index, kg/m^2^ | 24.4±2.9 | 24.2±2.1 | 24.0±3.0 | 24.9±3.9 |  | *F*=0.33 | 0.806 |  |
| Underwent MRI before, yes/no | 12/8 | 10/10 | 9/11 | 4/15 |  | *X^2^*=6.43 | 0.093 |  |
| **Study 2** | Healthy Controls | | Bipolar Patients | |  | Group difference | | |
|  | No-Stress  (N=20) | Stress  (N=20) | No-Stress  (N=16) | Stress  (N=20) |  | Test-statistic | p-value |  |
| Age, years | 40.7±7.4 | 38.6±7.9 | 42.4±8.1 | 39.8±8.6 |  | *F*=0.70 | 0.555 |  |
| Education^¥^, level obtained | 6 (4-7) | 6 (4-6) | 6 (5-7) | 6 (5-7) |  | *F*=1.01 | 0.396 |  |
| Handedness, right/left/both | 18/2/0 | 19/1/0 | 14/2/0 | 17/3/0 |  | *X^2^*=1.15 | 0.766 |  |
| Smoking, yes/no | 3/17 | 3/17 | 4/12 | 4/16 |  | *X^2^*=0.81 | 0.848 |  |
| Body Mass Index, kg/m^2^ | 25.9±3.8 | 25.6±3.6 | 26.0±3.1 | 25.5±3.4 |  | *F*=0.08 | 0.970 |  |
| Underwent MRI before, yes/no | 15/5 | 9/11 | 7/9 | 14/6 |  | *X^2^*=6.28 | 0.099 |  |

*Note.* The descriptive characteristics of patients and controls in either condition were compared for both studies. All values represent means and standard deviations for continuous variables, and either medians and interquartile range (^¥^) or frequencies for categorical variables. Education represents the highest level of education attained in the Dutch system (Study 1: 1-8, Study 2: 1-7).

**Supplementary Table 2** Eigenvector centrality change of brain regions within the dorsal attention network for controls in the stress condition compared to the no-stress condition.

| **Healthy Controls** | Stress-group relative to no-stress group | |  | | Mixed Effects Model  Stress vs. no-stress | |  |
| --- | --- | --- | --- | --- | --- | --- | --- |
|  | Centrality z-score Mean (±SD) | |  | | Time×Condition | |  |
|  | RS2 | RS3 | |  | | RS2-RS3 | |
| **Brainnetome Regions** |  |  | |  | |  | |
|  |  |  | |  | |  | |
| 7 | -0.368 (±1.26) | -0.213 (±1.19) | |  | | *F*=0.32 *p*=0.621 | |
| 8 | -0.182 (±0.90) | 0.000 (±0.97) | |  | | *F*=0.57 *p*=0.569 | |
| 25 | 0.280 (±1.43) | 0.076 (±0.91) | |  | | *F*=0.46 *p*=0.569 | |
| 55 | -0.373 (±1.23) | -0.173 (±1.39) | |  | | *F*=0.46 *p*=0.569 | |
| 56 | -0.382 (±0.91) | -0.086 (±1.12) | |  | | *F*=1.32 *p*=0.532 | |
| 63 | -0.016 (±1.30) | 0.060 (±0.77) | |  | | *F*=0.89 *p*=0.798 | |
| 64 | -0.009 (±0.79) | 0.204 (±0.78) | |  | | *F*=1.21 *p*=0.532 | |
| 85 | 0.189 (±1.04) | -0.023 (±0.92) | |  | | *F*=0.67 *p*=0.569 | |
| 86 | -0.050 (±0.85) | 0.206 (±0.87) | |  | | *F*=1.09 *p*=0.532 | |
| 92 | 0.079 (±0.94) | 0.387 (±1.22) | |  | | *F*=1.17 *p*=0.532 | |
| 97 | 0.162 (±0.92) | -0.026 (±1.19) | |  | | *F*=0.55 *p*=0.569 | |
| 98 | 0.287 (±1.02) | 0.504 (±1.08) | |  | | *F*=0.59 *p*=0.569 | |
| 125 | **-0.711 (±1.67)** | **0.250 (±0.94)** | |  | | ***F*=9.09 *p*=0.028** | |
| 126 | -0.536 (±1.15) | 0.124 (±0.22) | |  | | *F*=1.84 *p*=0.448 | |
| 127 | -0.198 (±0.87) | 0.104 (±0.87) | |  | | *F*=1.85 *p*=0.448 | |
| 128 | -0.051 (±0.99) | 0.322 (±0.67) | |  | | *F*=2.62 *p*=0.343 | |
| 129 | **-0.470 (±1.28)** | **0.261 (±0.79)** | |  | | ***F*=8.09 *p*=0.028** | |
| 130 | -0.306 (±1.10) | -0.045 (±1.17) | |  | | *F*=1.00 *p*=0.532 | |
| 133 | **-0.444 (±1.09)** | **0.482 (±0.86)** | |  | | ***F*=11.5 *p*=0.014** | |
| 134 | -0.318 (±0.94) | 0.295 (±0.98) | |  | | *F*=5.42 *p*=0.094 | |
| 136 | -0.037 (±0.85) | 0.217 (±0.86) | |  | | *F*=0.92 *p*=0.532 | |
| 139 | 0.211 (±1.17) | 0.190 (±0.92) | |  | | *F*=0.01 *p*=0.940 | |
| 140 | -0.019 (±0.94) | 0.626 (±1.09) | |  | | *F*=5.09 *p*=0.096 | |
| 148 | **-0.427 (±1.03)** | **0.309 (±0.78)** | |  | | ***F*=8.31 *p*=0.028** | |
| 150 | **-0.515 (±0.97)** | **0.286 (±1.01)** | |  | | ***F*=12.0 *p*=0.014** | |

*Note.* Healthy controls (HCs) in the stress condition show an increase over time in centrality *z*-scores for several brain regions within the dorsal attention network (DAN). The *z*-scores represent values of HCs in the stress condition relative to the distribution of HCs in the no-stress condition. All *p*-values are corrected for multiple comparisons using the false discovery rate. RS1=pre-stress exposure, RS2=20 min. post-exposure, RS3=90 min. post-exposure.

**Supplementary Table 3.** Within- and between-network connectivity change for the dorsal attention network of healthy controls in the stress condition compared to controls in the no-stress condition.

| **Healthy Controls** | Stress-group relative to no-stress group | |  | Mixed Effects Model  Stress vs. no-stress | |
| --- | --- | --- | --- | --- | --- |
|  | Centrality z-score Mean (±SD) | |  | Time×Condition | |
|  | RS2 | RS3 |  | RS2-RS3 | |
| **DMN-** |  |  |  |  |  |
|  |  |  |  |  |  |
| DMN | 0.064 (±1.04) | 0.206 (±1.14) |  | *F*=0.39 | *p*=0.626 |
| FPN | 0.032 (±0.78) | 0.462 (±1.18) |  | *F*=1.73 | *p*=0.220 |
| DAN | -0.046 (±0.82) | 0.361 (±1.16) |  | *F*=2.78 | *p*=0.232 |
| VAN | -0.048 (±0.82) | 0.273 (±1.09) |  | *F*=0.40 | *p*=0.274 |
| VIS | -0.014 (±0.81) | 0.494 (±1.19) |  | *F*=3.95 | *p*=0.220 |
| SMN | -0.033 (±0.93) | 0.255 (±1.23) |  | *F*=1.36 | *p*=0.346 |
| DGM | 0.262 (±1.22) | 0.282 (±1.13) |  | *F*=0.01 | *p*=0.934 |

*Note.* Healthy controls (HCs) in the stress condition show no change over time in within- (i.e. DAN-DAN) and between-network (e.g. DAN-DMN, DAN-FPN, etc.) connectivity z-scores of the dorsal attention network (DAN) between RS2 and RS3, relative to HCs in the no-stress condition. The *z*-scores represent values of HCs in the stress condition relative to the distribution of controls in the no-stress condition. All *p*-values are corrected for multiple comparisons using the false discovery rate. RS1=pre-stress exposure, RS2=20 min. post-exposure, RS3=90 min. post-exposure, DMN=default-mode network, FPN=frontoparietal network, VAN=ventral attention network, VIS=visual network, SMN=sensorimotor network, DGM=deep grey matter.
